# Corrigendum

**DOI:** 10.1002/ehf2.13456

**Published:** 2021-06-04

In the paper by Izumida *et al*.,^1^ the last author's name has been misspelt.

It should be “Koichiro Kinugawa” as per the corrected author list below:

Toshihide Izumida, Teruhiko Imamura, Makiko Nakamura, Nobuyuki Fukuda and Koichiro Kinugawa.

The online version has been modified to reflect the change.
